# Operating Room Black Box (ORBB): Examining Nurses’ Perceptions in a Surgical Setting

**DOI:** 10.1177/15533506251383336

**Published:** 2025-09-23

**Authors:** Pria Nippak, Victoria Ross, Housne Begum, Kimberley Okafor, Mya Rana-Nippak, Stanley J Hamstra, Markku Nousianinen

**Affiliations:** 1 Health Services Management, Ted Rogers School of Management, 7984 Toronto Metropolitan University, Toronto, ON, Canada; 2 Department of Surgery, Sunnybrook Research Institute, 7938 University of Toronto, Toronto, ON, Canada; 3 Holland Bone and Joint Program, Sunnybrook Health Sciences Centre, Toronto, ON, Canada

**Keywords:** operating room black box, surgical safety, nurse perceptions, patient safety, surgical complications, technology adoption, OR workflow

## Abstract

**Background:**

Despite numerous efforts to improve surgical safety, adverse events and serious surgical complications are still common. This cross-sectional study at a tertiary hospital in Ontario, Canada, aimed to examine nurses’ perceptions, awareness, comfort, and readiness to use Operating Room Black Box (ORBB) technology, implemented to reduce surgical errors.

**Methods:**

A mixed method was used and data was collected through a 14-item questionnaire in summer 2022.

**Results:**

Among 50 nurse participants, nurses with work experience <20 years had higher overall mean scores on 9 questions than nurses working >20 years. The majority (88.0%) had no prior ORBB experience but somewhat agreed that ORBB had the potential to improve the safety culture in the operating room.

**Conclusion:**

Overall, nurses demonstrated positive attitudes towards ORBB technology, indicating its potential to enhance safety culture, team communication, teamwork, situational awareness, feedback on performance, the debriefing process, transparency, and lead to technological advancements in healthcare.

## Background

Extensive efforts to enhance surgical safety have been undertaken, yet the incidence of adverse events and patient harm during surgery remains substantial.^1,2^ Serious surgical complications affect a significant proportion of the more than 200 million operations conducted globally each year, with human factors or system errors accounting for approximately half of these complications deeming them potentially preventable.^3-5^ A significant portion of surgical errors are a result of adverse events in the operating room (OR), and no individual, regardless of their clinical competence, is immune from it.^3,6-8^ Even prior to the surgical intervention stage, diagnostic errors occur across various disciplines and throughout all phases of care, with contributing factors frequently linked to communication breakdown and provider clinical decision-making.^9^ Thus, human error pervasively contributes to issues along the continuum of care. The ORBB technology, which is similar to aviation black box flight recorders, is intended to capture synchronized information during surgical procedures in a systematic and impartial manner,^3,4,10,11^ providing a documented account of human errors and adverse events.

The ORBB technology can be used to objectively and precisely document a clinical procedure in the OR (e.g., team performance, resiliency supports, and interpersonal communications), allowing for more prospective evaluation and purposeful retrospective case review, and can inform better technical improvement and improve intraoperative error detection (e.g., near misses and distractions).^3,12,13,18^ Awareness of factors influencing patient care can lead to interventions (e.g., noise control during certain phases of surgery) that are specific to the surgical team environment, thereby optimizing patient safety within that context.^13,14^ Furthermore, the ORBB technology can help to reduce postoperative errors, reveal implicit behaviors that might otherwise go unnoticed without recording, identify potential causes that compromise patient health and safety, and improve patient outcomes and quality of care.^8,11,14,15^ ORBB evaluates trainees’ performance objectively, emphasizing what works and what needs to be improved.^12^ Furthermore, it allows for objective monitoring of trainees’ progress throughout training, promoting competency-based education.^12,16^ Moreover, ORBB technology can elucidate intraoperative factors that have a negative impact on patient care (e.g., errors and adverse events) such as medication errors, which is one of the most common forms of medical errors.^17^ It can also significantly improve the method of professional development currently used by perioperative nurses and surgeons because they can benefit professionally from reviewing OR footage and identify opportunities for improvement.^11,13,18^ Based on the many benefits, a large urban teaching hospital in Toronto is in the preliminary stages of a 3-year black box implementation study. Successful implementation of new technology is heavily reliant on successful adoption.^19^ Stakeholder acceptance may be the single most important deciding factor in whether a new technology succeeds or fails.^20,21^ Studies have shown that increasing stakeholder knowledge and awareness prior to new technology adoption can foster better user reception.^22,23^ One of these key stakeholders are nurses, who play a critical role in the healthcare workforce and more specifically in surgical settings where the ORBB technology is intended to be used. For these reasons the current investigation sought to engage nurse feedback in an effort to promote nurse buy-in, a crucial component to reducing potential barriers to successful adoption of new technology like ORBB.^24-26^ In the current investigation, the initial aim of increased awareness of ORBB technology was achieved through engagement and assessment of how the technology can be used to improve surgical processes through the use of a survey tool designed to solicit nurse feedback. Furthermore, this was a pilot study that was part of a larger study, and perceptions of ORBB were being evaluated on one unit, not the entire hospital. The study aimed to assess nurses’ awareness, comfort, and readiness to use ORBB technology, as well as to explore how to use ORBB technology most effectively in the surgical setting. The research team also identified a knowledge gap in the lack of a tool to examine nurses’ perceptions of ORBB technology in a surgical setting, which this study sought to address.

## Materials and Methods

The study was conducted at a prominent academic health sciences center in Canada. This tertiary center is known for its advanced research and patient care and is affiliated with a university serving as a major teaching hospital. This healthcare center is in the preliminary stages of a 3-year black box implementation study. The research team planned to install 2 black boxes, one in OR03 and one in OR01, equipped with cameras, encoders, a touchscreen, and microphones to gather data during surgical procedures. The data collected was transmitted to the IT Closet in OR03 and then securely transferred to Surgical Safety Technology’s (SST’s) data center for analysis. There was a collaboration between the tertiary hospital and another local hospital indicating a coordinated effort in adopting this technology. In this current study, we wanted to look at nurses’ perceptions of ORBB technology in surgical settings, specifically focusing on identifying the key benefits that nurses believe ORBB technology can offer in enhancing surgical practices.

### Participant Recruitment

The study subjects consisted of nurses at a tertiary hospital who were introduced to the ORBB during a team meeting, as part of the ORBB implementation initiative. This cross-sectional study employed a mixed-methods approach, combining quantitative and qualitative methods. While the study design was primarily quantitative, by incorporating a questionnaire as the main research tool, qualitative insights were also gathered through an open-ended question in the questionnaire. All nurses on the unit within the hospital were identified and invited to participate in the research project; the nursing champion assumed the role of recruiting participants, and introduced the survey via email. Nurses were invited to participate in the study and the questionnaire was distributed to them during team staff meetings. Completion of the questionnaire implied consent.

### Ethical Considerations

Given the Quality Improvement (QI) nature of the project, the urban teaching hospital site completed an Ethics Review using the Self Assessment Tool (ER-SAT), which confirmed the quality improvement status of the project. The Research Ethics Board approved the study protocol on Monday September 19th, 2022 (REB 2022-398) to allow the data collected to be used for secondary publication purposes.

### Measurement Instruments and Procedure

The study employed a mixed methods approach, including quantitative and qualitative methods, to examine nurses’ perceptions towards ORBB technology and its potential to improve processes in the operating room (OR) from the nurses’ perspective. The study was primarily quantitative, aiming to identify the most significant potential benefits that nurses believe ORBB technology can achieve in a surgical setting at a tertiary hospital. The primary research methodology was a questionnaire modified from existing literature to fit the context of ORBB technology.^3,4,7,11,14^ The study questionnaire was distributed to nurses working on a single unit at the tertiary hospital in the Fall of 2022. The nursing champion identified 50 nurses as potential participants. All 50 consented to participate in the study and completed the questionnaire. Moreover, no additional sampling procedures were required because all 50 nurses consented on the unit were recruited and participate in the pilot study.

The questionnaire was distributed by the nursing champion to both Registered Nurses (RNs) and Registered Practical Nurses (RPNs). Prior to distribution, nurses were informed about the survey in team staff meetings and were told that completion of the survey would serve as their consent. Recruitment began on June 10, 2022, and ended on June 17, 2022. The nursing champion distributed questionnaires exclusively to nurses following 2 consecutive Friday team staff meetings at the tertiary hospital. The questionnaire consisted of 9 questions about nurses’ perspectives on ORBB technology, expressed on a 7-point Likert scale ranging from 1 (strongly disagree) to 7 (strongly agree). The 9 questions, as shown in Appendix 1, were Q1: to improve operating room safety culture; Q2: to improve operating room team communication; Q3: to improve teamwork and collaboration in the operating room; Q4: to improve situational awareness of the surgical team; Q5: to receive appropriate and timely feedback on performance; Q6: to improve the debriefing process; Q7: to identify potential intraoperative safety risks (e.g., cognitive and auditory distractions, door opening, non-case-related conversations, repeated turnover of personnel); Q8: to increase transparency in the clinical setting; and Q9: to lead to technological advancements in health care. A score of 1 indicated strongly disagree; a score of 2 indicated disagree; a score of 3 indicated somewhat disagree; a score of 4 indicated neutral; a score of 5 indicated somewhat agree; a score of 6 indicated agree; and a score of 7 indicated strongly agree. As a result, an item score of 5 to 7 indicated a positive response. A total domain score of 35 or higher indicated more positive attitudes toward the use of ORBB technology.

The final questionnaire contained 14 items constructed by the research team based on previous studies to capture nurses’ perceptions of ORBB technology and how it could be used to improve processes in the OR.^3,4,11,14,27-29^ The responses to 9 of the 14 questions were based on a 7-point Likert scale. The second survey section included an open-ended question that invited nurses to provide a written response in the free-text box field about their perceptions toward the use of ORBB technology, and how it could be used to improve processes in the OR.

### Statistical Analysis

The reliability and validity of the constructs were calculated. Constructs were combined to perform factor analysis. Factor loading depicts the relationship between each item and the associated constructs.^30^ Principal axis factoring (PAF) with Varimax rotation was used for all 9 variables. All variables were loaded into a single component and were calculated loading factors. Convergent validity was assessed using the outer loadings of the indicators. Moreover, internal validity was assessed using Cronbach’s alpha. Cronbach’s alpha (α) > 0.70 is typically considered to be significant and an “acceptable” level of reliability.^31^

Additionally, the content validity of the questionnaire was established by reviewing several works of literature on the subject, modifying the questionnaire based on feedback and comments from the 5 colleagues to whom the questionnaire was initially piloted, and incorporating the expert opinion of the research team members.^3,11,14,27-29^

Descriptive statistics were computed for all baseline demographic characteristics. Characteristics were presented as n (number of individuals), frequencies, and percentages (%), as appropriate. Chi-Square tests were used where appropriate to test the relationships between construct responses and variables (e.g., gender, role in the OR, work experience, and ORBB experience).

Furthermore, the Chi-Square Test was corrected, resulting in the Fisher-Freeman-Halton Exact Test being used to determine the association between the variables. The Cronbach alpha was used to assess internal validity.^32^ Cronbach alpha values between 0.7 and 0.9 are considered satisfactory.^33^ The outer loadings of the indicators were used to assess convergent validity.^32^ The outer loadings should be 0.708 or higher to help establish convergent validity on the construct.^33^ All paper data records from participants were converted to a password-protected Excel database (version 2010) and exported into SPSS Statistics (version 25; IBM Corporation). The results of the survey were analyzed using SPSS Statistics for descriptive statistics. The level of statistical significance was set at *P* < 0.05.

## Results

### Participants Characteristics

In Fall 2022, all 50 nurses consented on the unit were recruited, representing the entire nursing population within the unit. The 50 participants comprised mostly females (82.0%), and only 8.0% individuals who did not indicate their gender (Table 1). With regards to role in the OR, the highest number of nurses (44.0%) were both scrub and circulating nurses followed by scrub nurses (32.0%), circulating nurses (20.0%), and 4.0% did not indicate their role in the OR. Most of the nurses (42%) work experience 21 years followed by < 10 years (32%) and 20-29 years (18%). When they were asked about ORBB experience, Majority (88.0%) nurses did not have ORBB experience.

**Table 1.** Sociodemographic Characteristics of the Respondents (n = 50)

Variable	*n* (%)
Gender	
Male	5 (10.0)
Female	41 (82.0)
Other/undisclosed	4 (8.0)
Role in the OR	
Scrub nurse	16 (32.0)
Circulating nurse	10 (20.0)
Dual roles	22 (44.0)
Undisclosed	2 (4.0)
Work experience (years)	
<10	16 (32.0)
10-19	21 (42.0)
20-29	9 (18.0)
30-39	1 (2.0)
≥40	1 (2.0)
Undisclosed	2 (4.0)
ORBB experience	
No	44 (88.0)
Yes	3 (6.0)
Undisclosed	3 (6.0)
Total	50 (100)

### Scale Reliability

Table 2 describes the reliability and validity of the constructs. For each of the 9 variables, Principal axis factoring (PAF) with Varimax rotation was used, and factor loadings were all above 0.8. Convergent validity using the outer loadings of the indicators were all >0.708, ranging from 0.85 to 0.94, which established the convergent validity of the construct.^33^ Also, the Corrected Item-Total Correlation for the 9 individual components of the multi-item scale were all >0.30, ranging from 0.833 to 0.922; this indicated that these items belong on the scale because they demonstrated high internal consistency, and all measured the same construct. Moreover, internal validity. Cronbach’s alpha was 0.973 for 9 items on the questionnaire (Table 2). This value indicated that the scale had high levels of inter-item reliability. Because Cronbach’s Alpha (α) was α > 0 .70.^33^

**Table 2.** Construct Reliability

Construct*	Factor loading	Correlated item-total correlation	Cronbach alpha
Q1	.845	.833	.973
Q2	.880	.867	
Q3	.900	.886	
Q4	.904	.892	
Q5	.914	.901	
Q6	.930	.915	
Q7	.938	.922	
Q8	.857	.844	
Q9	.905	.892	

*Q1: safety culture, Q2: team communication, Q3: teamwork, Q4: situational awareness, Q5: feedback, Q6: debriefing process, Q7: identifying safety risks,Q8: transparency, and Q9: technological advancements in healthcare.

### Perceptions Towards Black Box

To evaluate the perceived impact of ORBB technology on various aspects of operating room performance (safety culture, team communication, teamwork, situational awareness, feedback, debriefing process, identifying safety risks, transparency, and technological advancements in healthcare), participants rated their agreement with statements on a Likert scale ranging from 1 (Strongly Disagree) to 7 (Strongly Agree). The primary focus was to assess overall perceptions of ORBB technology, primarily captured in Part 2 of the questionnaire, with construct summary measures derived from 9 researcher-generated questions (Table 3). Firstly, gender was significantly associated with the perceived potential of ORBB in several aspects. Women were more likely to agree that ORBB can help improve the safety culture (*P* = .012), improve situational awareness (*P* = .010), receive feedback on performance (*P* = .015), improve the debriefing process (*P* = .015), potential intraoperative safety risks (*P* = .005), and lead to technological advancements (*P* = .030) (Table 3). However, perceptions did not significantly vary by role in the OR, work experience, or ORBB experience. (Table 3).

**Table 3.** *P* Values for Likelihood Ratio and Fisher-freeman-halton Exact Test

Construct	Gender		Role in the OR		Work experience (years)		ORBB experience	
	*P* Value							
	Likelihood ratio	Fisher-freeman-halton exact test	Likelihood ratio	Fisher-freeman-halton exact test	Likelihood ratio	Fisher-freeman-halton exact test	Likelihood ratio	Fisher-freeman-halton exact test
Q1	.012*	.012*	.423	.576	.192	.128	.416	.235
Q2	.203	.203	.945	.953	.921	.942	.195	.195
Q3	.230	.138	.633	.759	.584	.452	.051	.080
Q4	.010*	.010*	.623	.619	.527	.462	.116	.116
Q5	.015*	.015*	.674	.824	.077	.073	.092	.092
Q6	.015*	.015*	.716	.874	.424	.293	.054	.085
Q7	.005**	.005*	.369	.572	.070	.048	.624	.374
Q8	.196	.078	.302	.398	.352	.245	.720	.355
Q9	.030*	.030*	.328	.488	.255	.183	.111	.111

Level of significance at <0.05*, 0.005**, Q1: safety culture, Q2: team communication, Q3: teamwork, Q4: situational awareness, Q5: feedback, Q6: debriefing process, Q7: identifying safety risks,Q8: transparency, and Q9: technological advancements in healthcare.

### Overall Participant Responses to Questionnaire Items

In Table 4, participant responses to the 9 questionnaire items irrespective of gender, role in the OR, work experience, and ORBB experience were examined. Overall, most participants had positive perceptions toward ORBB. With regards to improving the safety culture in the operating room (Q1), 58.0% (n = 29) agreed (ranging from somewhat agree to strongly agree); 28.0% (n = 14) disagreed (ranging from somewhat agree to strongly agree); and 14.0% (n = 7) were neutral. The highest percentage of respondents (n = 13, 26.0%) somewhat agreed. With regards to improving team communication in the operating room (Q2), 40.0% (n = 20) agreed; 42.0% (n = 21) disagreed; and 14.0% (n = 7) were neutral. The highest percentage of respondents (n = 9, 18.0%) agreed. With regards to improving teamwork and collaboration in the operating room (Q3), 48.0% (n = 24) agreed; 34.0% (n = 17) disagreed; and 18.0% (n = 9) were neutral. The highest percentage of respondents (n = 10, 20.0%) strongly agreed.

**Table 4.** Overall Participant Responses to the Questionnaire

Construct	*n* (%)							
	1 strongly disagree	2 disagree	3 somewhat disagree	4 neutral	5 somewhat agree	6 agree	7 strongly agree	Total (Missing responses)
Q1	6 (12.0)	5 (10.0)	3 (6.0)	7(14.0)	13 (26.0)	10(20.0)	6 (12.0)	50
Q2	6 (12.0)	8 (16.0)	7 (14.0)	7(14.0)	5 (10.0)	9 (18.0)	6 (12.0)	48 (2)
Q3	8 (16.0)	5 (10.0)	4 (8.0)	9(18.0)	9 (18.0)	5 (10.0)	10 (20.0)	50
Q4	7 (14.0)	4 (8.0)	4 (8.0)	8(16.0)	14 (28.0)	7(14.0)	6 (12.0)	50
Q5	3 (6.0)	6 (12.0)	5 (10.0)	5(10.0)	13 (26.0)	9 (18.0)	9 (18.0)	50
Q6	5 (10.0)	6 (12.0)	4 (8.0)	7(14.0)	13 (26.0)	8 (16.0)	7 (14.0)	49 (1)
Q7	7 (14.0)	6 (12.0)	4 (8.0)	6(12.0)	11 (22.0)	9 (18.0)	7 (14.0)	50
Q8	7 (14.0)	2 (4.0)	3 (6.0)	7(14.0)	14 (28.0)	10(20.0)	7 (14.0)	50
Q9	4 (8.0)	5 (10.0)	4 (8.0)	5(10.0)	8 (16.0)	14(28.0)	9 (18.0)	49 (1)

Q1: safety culture, Q2: team communication, Q3: teamwork, Q4: situational awareness, Q5: feedback, Q6: debriefing process, Q7: identifying safety risks, Q8: transparency, and Q9: technological advancements in healthcare.

With regards to improving the situational awareness of the surgical team (Q4), 54.0% (n = 27) agreed; 30.0% (n = 15) disagreed; and 16.0% (n = 8) were neutral. The highest percentage of respondents (n = 14, 28.0%) agreed. With regards to receiving appropriate and timely feedback on performance (Q5), 62.0% (n = 31) agreed; 28.0% (n = 14) disagreed; and 10.0% (n = 5) were neutral. The highest percentage of respondents (n = 13, 26.0%) somewhat agreed. With regards to improving the debriefing process (Q6), 56.0% (n = 28) agreed; 30.0% (n = 15) disagreed; and 14.0% (n = 7) were neutral. The highest percentage of respondents (n = 13, 26.0%) somewhat agreed. With regards to identifying potential intraoperative safety risks (Q7), 54.0% (n = 27) agreed; 30.0% (n = 15) disagreed; and 12.0% (n = 6) were neutral. The highest percentage of respondents (n = 11, 22.0%) somewhat agreed. With regards to increasing transparency in the clinical setting (Q8), 62.0% (n = 31) agreed; 24.0% (n = 12) disagreed; and 14.0% (n = 7) were neutral. The highest percentage of respondents (n = 14, 28.0%) somewhat agreed. Lastly, with regards to leading to technological advancements in health care (Q9), 62.0% (n = 31) agreed; 26.0% (n = 13) disagreed; and 10.0% (n = 5) were neutral. The highest percentage of respondents (n = 14, 28.0%) agreed. Furthermore, for each of the 9 questionnaire items, a small percentage of respondents strongly disagreed, ranging from 6.0% to 16.0%.

### Participant Responses to Open-Ended Questions

The final item (Question 10) was an open-ended question inviting a written response regarding the potential uses and/or anticipated benefits of ORBB technology for nurses in a surgical setting at the tertiary hospital. Specifically, how nurses believe this technology can be used to improve processes in the OR. This question was completed by 9 of 50 (18%) participants, resulting in 41 incomplete sections for this aspect of the survey. Among the 9 participants who completed this section, some expressed positive attitudes toward the use of ORBB technology, while others expressed negative attitudes, and others provided neutral responses (Participant 3). ORBB, according to participants, can be “seen as a way to punish team members” and forces employees to “obligate and agree or [they] will be barred from working in the [operating] room” (Participant 1; Participant 2). Participants were also “worried about [the] potential use of materials for litigation purposes” and how “this technology is detrimental to the surgical team” (Participant 2; Participant 5). According to Participant 8, ORBB is “likely to cause many nurses to change their behaviours in the OR” and become “anxious and distracted about potential repercussions, which may negatively impact patient care.” It was also stated that there could be “potential misinterpretation of video, audio, or the implications to patients” and “there is no need for it [ORBB] in OR” (Participant 2; Participant 6). However, participants also articulated more positive feelings about ORBB. Participants expressed that “black box has the potential to improve quality of care of staff performance” as well as “address patient safety and efficiency in the OR” (Participant 4; Participant 7). Participants also commented that “there appear to be several advantages to using black box technology” and it has “the potential to standardize procedures in a way that will improve care” (Participant 4; Participant 9). Furthermore, participants expressed that “for this technology to be more widely adopted, the benefits of the black box must be balanced with effective policies that protect the interests of both nurses and their patients,” and staff “have to feel safe from concerns” (Participant 5; Participant 9).

## Discussion

This study examined nurses’ perceptions of novel surgical application ORBB technology at an academic health center located in Canada. The primary focus was to understand its potential to improve surgical processes and engage nurses in quality improvement measures. The results of the study highlight the influence of gender on nurses’ perceptions to ORBB technology. These findings underline a critical gap in Canadian literature by showcasing the various factors that affect perceptions of ORBB technology.

This study found significant gender differences in perceptions of ORBB technology with male participants (10.0%) showing fewer positive attitudes compared to female participants (82.0%). Given that nursing is a female-dominated profession, there is a lack of research focusing on gender-based or sex differences in nurses’ perceptions of technology^34^ as females are largely overrepresented in studies. However, Korte et al.^35^ and their team showed that male nurses display more technical readiness compared to female nurses. These gender differences between male and female nurses could be due to the increase of technology-based educational tools within the nursing department aimed to improve healthcare operations among nurses.^36^ espite the gap in the literature on gender differences among nurses’ perceptions of technology, the findings of our study indicate a need for future research with proportional gender representation to gain further insights.

While the nurses’ role did not produce statistically significant findings, the nursing role trends observed were consistent with the findings from other similar studies that reported significant effects.^37^ In both studies, scrub nurses reported the most positive perceptions of ORBB technology, relative to dual role and circulating nurses. Zuzelo et al,^37^ suggested that these specialized nurses may be more accustomed to new technologies given their roles. Similarly, while statistically insignificant, the impact of years of nursing experience on ORRB attitudes demonstrated trends whereby the most experienced nurses reported the most positive attitudes toward ORBB technology. These findings contrast with previous studies indicating the opposite finding with younger healthcare professionals reported more positive attitudes around the use of new technologies.^38,39^ Several studies have reported a correlation between resistance to change that is positively correlated with increased experience.^23,39,40^ Other studies have shown that targeted education, specific training, and mentoring may aid in the implementation of ORBB technology among circulating and dual-role nurses.^23,26^ Additionally, studies suggest that constant support and practice opportunities could enhance the transition to this new technology.^23,40-43^

Despite a lack of statistical significance, an interesting relationship between prior exposure to ORBB technology and favorable perceptions was noted warranting further investigation with a larger sample size. Nurses who had no prior exposure to ORBB technology reported higher mean scores, relative to nurses with prior exposure. This finding aligns with previous studies that have used the technology acceptance model (TAM), where individuals are more likely to be receptive and have favorable perceptions of new technology because it is perceived as innovative.^44-46^ The perceived usefulness and ease of use of this technology could have influenced the participants’ perception of ORBB technology despite not having prior exposure.

Another finding identified in this study was nurses’ lack of readiness for change. This was exemplified through nurses’ statements that ORBB technology would be detrimental to the surgical team; result in potential misinterpretation, distractions, and surgical team members changing their behaviour; and ORBB technology implementation is simply unnecessary. These perceptions and their impact on implementation require further investigation.

It is important to devise a thorough education and engagement strategy before implementing ORBB technology^47^ to maintain the emphasis on continuous quality improvement. As well, there should be clear information provided about data management, usage, and legal aspects to alleviate healthcare professionals’ fears.^48^ As the rollout of this ORBB technology continues, it would be beneficial to repeat the survey 6 months after ORBB implementation to gauge nurses’ attitudes and readiness more accurately, given that at that point, they will have more engagement with ORBB. The findings from a follow-up survey would provide feedback that could be used to adjust or improve ORBB processes, to ensure continuous quality improvement. Additionally, as ORBB technology is rolled out to other surgical departments across the hospital it will allow for the opportunity to expand the sample, providing access to more robust quantitative and qualitative data.

## Limitations

The lack of response on all open-ended questions (only 9 out of 50 responses in the open-ended question) may represent a non-response bias or a form of resistance or a lack of familiarity and knowledge, in which the nurse participants who did not respond may have a negative or impartial attitude toward the questionnaire or operative recording in general. The quantitative approach of this ORBB study also has limitations in terms of the amount of detail and individual variation that it can capture. Future qualitative studies could be conducted to further elucidate healthcare professionals’ perspectives on ORBB technology. The small sample size is another limitation of this study, which only allowed for large differences to be detected between groups. There was also a disproportionate male-to-female participant ratio, with 41 female-identified participants compared to 5 male-identified participants, while this aligns with other study findings, gender differences could not be fully examined because of the unequal sample sizes between the 2 genders.^49^ This was a single-hospital study involving a specific population of interest, professionally and geographically at a single point in time; the opinions expressed in this study may not be representative of the opinions expressed by nurses at other institutions. Though the findings of this study may not be broadly generalizable across other hospital settings, they may serve as a foundation for future research into nurses’ concerns about operative recording and strategies to encourage the uptake of this novel tool.

Also, future research that intends to conduct a similar study should recognize that Registered Nurses can work dual roles in the OR. An oversight in this survey was that it only provided the option for participants to select their role in the OR as “Scrub nurse” or “Circulating nurse.” However, nurses who worked both roles in the OR indicated their dual role status by circling both roles on the questionnaire; this improved data accuracy by allowing dual role status to be acknowledged throughout the paper.

## Conclusion

Overall, the study findings showed that while there was general agreement on the potential benefits of using ORBB technology, there were also concerns and hesitations among nurses, particularly regarding data privacy, usage, and potential repercussions. Despite assurances of de-identification and data scrubbing, participants expressed anxiety and distraction as the key concerns around knowing they would be recorded. These findings suggest that nurses may be hesitant or resistant to adopting ORBB technology, which should be a consideration in the ORBB adoption plan.

## Appendix 1: Operating Room Black Box Questionnaire

### Operating Room Black Box (ORBB): Examining Nurses’ Perceptions in a Surgical Setting

Part I: Characteristics of Participating Nurses.

1. Gender
  a. Male
  b. Female
2. Role in the Operating Room
  a. Scrub nurse
  b. Circulating nurse
3. Work Experience (Years)
  a. < 10
  b. 10-19
  c. 20-29
  d. 30-39
  e. ≥40
4. ORBB Experience
  a. No
  b. Yes

### Part II: Your Views on Operating Room Black Box Technology

ORBB is a comprehensive surgical data recording system that includes several cameras, sensors, error analysis software (e.g., mobile applications), and microphones that are linked to a series of computers to capture and synchronize various sources of intraoperative visual and auditory data (e.g., distractions), patient physiology (e.g., vital signs), nontechnical and technical data, interpersonal and intrapersonal dynamics, and various environmental factors in the OR.

Please indicate your level of agreement with the following statements, where 1 = Strongly disagree and 7 = Strongly agree.
